# Cost-effectiveness of oxaliplatin and capecitabine in the adjuvant treatment of stage III colon cancer

**DOI:** 10.1038/sj.bjc.6603348

**Published:** 2006-10-10

**Authors:** S Eggington, P Tappenden, A Pandor, S Paisley, M Saunders, M Seymour, P Sutcliffe, J Chilcott

**Affiliations:** 1 School of Health and Related Research, University of Sheffield, Regent Court, 30 Regent Street, Sheffield, S1 4DA, UK; 2 Department of Clinical Oncology, Christie Hospital, Wilmslow Road, Manchester, M20 4BX, UK; 3 Cancer Research UK Clinical Centre, Cookridge Hospital, Leeds, LS16 6QB, UK

**Keywords:** oxaliplatin, capecitabine, adjuvant chemotherapy, colonic neoplasms, economic evaluation

## Abstract

For many years, the standard treatment for stage III colon cancer has been surgical resection followed by 5-fluorouracil in combination with folinic acid (5-FU/LV). Ongoing clinical trial evidence suggests that capecitabine and oxaliplatin (in combination with 5-FU/LV) may improve disease-free survival and overall survival when compared against 5-FU/LV alone in the adjuvant setting. This study evaluates the cost-effectiveness profiles of these two regimens in comparison to standard chemotherapy, using evidence from two international randomised controlled trials. Survival modelling techniques were employed to extrapolate survival curves from the two trials in order to estimate the long-term benefits of alternative treatment options over the remaining lifetime of patients. The health economic analysis suggests that capecitabine is expected to produce greater health gains at a lower cost than 5-FU/LV. Oxaliplatin in combination with 5-FU/LV is estimated to cost £2970 per additional QALY gained when compared to 5-FU/LV alone. Future research should attempt to elucidate uncertainties concerning the optimal roles of capecitabine and/or oxaliplatin in the adjuvant setting in order to achieve the maximum level of clinical benefit.

Cancer of the large bowel is the third most common cancer in the UK, after breast and lung cancer. In 2002, there were 29 547 new cases registered in England and Wales, which represents over 12% of all new cancers in the UK (Cooper *et al*, 2005; Welsh Cancer Intelligence and Surveillance Unit, 2005). Approximately two thirds of all newly diagnosed cases of colorectal cancer (CRC) occur within the colon, and around 26% of these are classified as stage III (Dukes' C), in which the cancer has spread to at least one lymph node (South West Cancer Intelligence Service, 1995). Approximately 50–60% of patients will suffer a recurrence of their disease (Benson *et al*, 2004).

Evidence suggests that patients with stage III colon cancer have a 5-year survival rate of between 25 and 60% (van Cutsem and Kataja, 2005). Until recently, the current standard treatment for patients with stage III colon cancer was surgical resection (with curative intent) followed by a 6-month course of adjuvant intravenous 5-fluorouracil in combination with folinic acid (5-FU/LV) for those patients deemed sufficiently fit to tolerate the adverse effects of chemotherapy (National Institute for Clinical Excellence, 2004a). Several 5-FU/LV regimens exist, each of which may vary in terms of efficacy and adverse event profiles. The once-weekly bolus QUASAR regimen is most commonly used in England and Wales, although there is variation between cancer centres.

More recently, treatment using oxaliplatin (in combination with 5-FU/LV) and capecitabine has been trialled in the adjuvant treatment of patients with stage III colon cancer. Oxaliplatin is an intravenously administered, water soluble, platinum-based cytotoxic compound that forms intra- and inter-strand adducts with deoxyribose nucleic acid (DNA) leading to cell damage. Oxaliplatin, in combination with 5-FU/LV, is licensed in the EU for the adjuvant treatment of stage III colon cancer after complete resection of the primary tumour, and for the treatment of metastatic CRC. Capecitabine is an orally administered, non-cytotoxic fluoropyrimidine carbamate, and is a precursor of 5-FU. Capecitabine is currently licensed in the EU for the adjuvant treatment of patients with stage III colon cancer following surgery and for first-line monotherapy in patients with metastatic CRC.

Within this study, we developed a health economic model to estimate the marginal cost-effectiveness of oxaliplatin plus 5-FU/LV and capecitabine in comparison to standard chemotherapy (5-FU/LV alone) in the treatment of patients with completely resected stage III colon cancer in England and Wales. The model synthesises the best available evidence on the costs and consequences resulting from the use of oxaliplatin and capecitabine to inform whether these therapies represent value for money for the NHS in England and Wales. This study was undertaken to inform the National Institute for Health and Clinical Excellence's (NICE) technology appraisal of oxaliplatin and capecitabine for the adjuvant treatment of stage III colon cancer (National Institute for Health and Clinical Excellence, 2006).

## METHODS

### Model structure and scope

We undertook a systematic review of evidence relating to the clinical effectiveness of capecitabine and oxaliplatin plus 5-FU/LV in comparison to 5-FU/LV in the adjuvant treatment of stage III colon cancer (Pandor *et al*, 2005). Three relevant trials were identified: (1) the Multi-Centre International Study of Oxaliplatin/5-FU and leucovorin in the Adjuvant Treatment of Colon Cancer (MOSAIC) (André *et al*, 2004), (2) The Xeloda™ Adjuvant Chemotherapy Trial (X-ACT) (Scheithauer *et al*, 2003; Twelves *et al*, 2005) and (3) the National Surgical Adjuvant Breast and Bowel Project (NSABP C-07) (Wolmark *et al*, 2005). Efficacy data from the NSABP C-07 trial were not mature at the time of writing and were presented only in abstract form, hence only clinical and resource use evidence from the MOSAIC and X-ACT studies were included in the health economic analysis. Both studies were multi-centre, international randomised controlled trials (RCTs). The MOSAIC study was designed to evaluate the adjuvant use of oxaliplatin in combination with infusional 5-FU/LV (FOLFOX4), compared with infusional 5-FU/LV alone (the LV5FU2 or de Gramont regimen) in patients with both stage II and stage III colon cancer. The primary efficacy end point within the MOSAIC trial was disease-free survival (DFS), whilst secondary end points included overall survival (OS), safety and long term adverse effects (André *et al*, 2004). The X-ACT study was designed to demonstrate that capecitabine monotherapy was at least equivalent to 5-FU/LV (Mayo Clinic regimen) in terms of DFS when administered as adjuvant treatment following surgery for stage III colon cancer. Secondary end points included relapse-free survival, OS, safety (including treatment toxicity), and quality of life (Scheithauer *et al*, 2003; Twelves *et al*, 2005). Table 1 details the four treatment regimens included in the health economic analysis.

**Figure 1:**
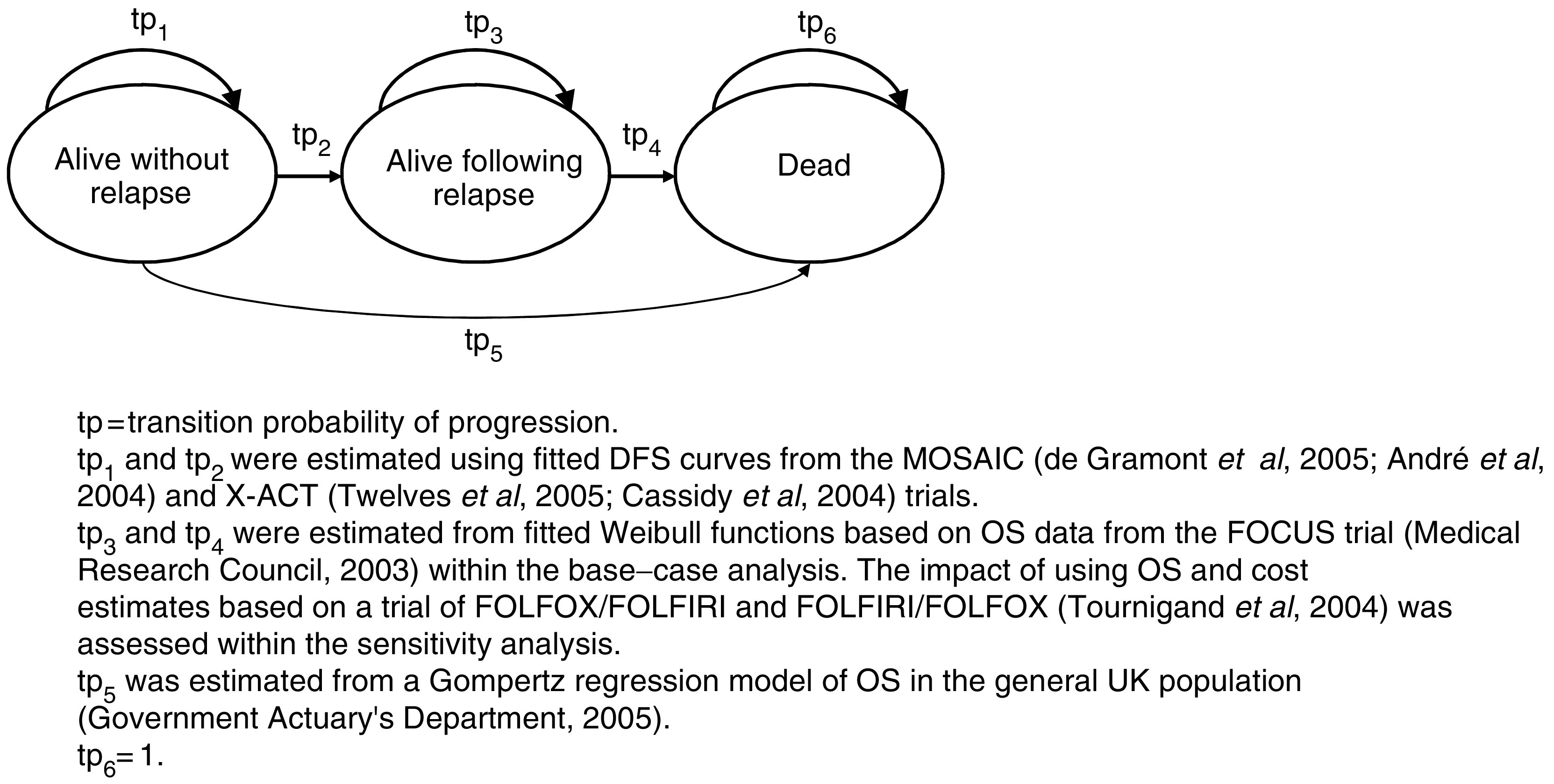
Schematic of health economic model.

A state transition model was developed using empirical DFS and OS curves reported within the MOSAIC trial (André *et al*, 2004; de Gramont *et al*, 2005) and the X-ACT trial (Cassidy *et al*, 2004; Twelves *et al*, 2005) to estimate the clinical and cost consequences of FOLFOX4 and capecitabine over the remaining lifetime of patients. State transition models are particularly useful for diseases or conditions whereby risk is ongoing over time, where events may occur more than once, and where the timing of events is important (Sonnenberg and Beck, 1993; Briggs and Sculpher, 1998). Clinically important events such as disease progression and death are modelled as transitions between health states. The primary health economic outcomes are the marginal cost per life-year gained (LYG) and the marginal cost per quality-adjusted life-year (QALY) gained for FOLFOX4 versus 5-FU/LV (de Gramont regimen), and for capecitabine *vs* 5-FU/LV (Mayo Clinic regimen). A simple schematic of the health economic model is presented in Figure 1.

The model is centred around three health states: alive without relapse, alive following relapse, and dead. Transitions between health states were derived from published survival curves using a 4-weekly cycle length. Costs and health outcomes were modelled according to the number of patients residing within each health state and the number of patients transiting between health states. The assumptions which underpin the health economic analysis are presented in Box 1.

The time-dependent probability of relapse was modelled by fitting parametric Weibull survivor functions to empirical DFS curves observed within the MOSAIC (André *et al*, 2004; de Gramont *et al*, 2005) and X-ACT (Cassidy *et al*, 2004; Twelves *et al*, 2005) studies using regression analysis. This form of extrapolative modelling was used to predict time-to-event outcomes where empirical trial data were censored. The model assumes that all relapses occur within five years following complete resection of the primary tumour, based upon evidence on the long-term follow-up of patients with stage III colon cancer undergoing adjuvant chemotherapy (Moertel *et al*, 1995). The expected survival of patients following relapse was modelled using a parametric Weibull survival model based on the experience of patients enrolled in the FOCUS trial (Maughan *et al*, 2005). The long-term survival of patients who do not relapse was modelled using a UK life table model (Government Actuary's Department, 2005); the model thus assumes that these patients have the same life expectancy as an age-matched population of individuals with no history of colon cancer.

### Valuation of health outcomes

Health utility scores were not collected within either the X-ACT or MOSAIC trials. Modelled survival estimates were adjusted to account for the patient's level of health-related quality of life using published colorectal cancer utility estimates. A utility score of 0.7 was assigned to patients receiving adjuvant treatment who experienced no significant serious adverse effects (Ness *et al*, 1999), while patients who suffered significant adverse events were assigned a utility score of 0.63 for the duration of the treatment course (Ness *et al*, 1999). Patients who remained disease-free following adjuvant treatment were assigned a utility score of 0.92 (Ramsey *et al*, 2000), while patients who relapsed were assigned a utility score of 0.24 (Ness *et al*, 1999). In line with recommendations from NICE at the time of the analysis, health outcomes were discounted at a rate of 1.5%.

### Resource use and costs

The health economic analysis was undertaken from the perspective of the UK National Health Service (NHS) and Personal Social Services (PSS). The model includes costs associated with drug acquisition and administration, pharmacy handling and dispensing, infusor pumps, examinations and tests, as well as hospitalisation resource use for the management of treatment-related toxicities. Data concerning chemotherapy usage, dose intensity and the costs of managing chemotherapy-related toxicities within the MOSAIC and X-ACT trials were made available by Sanofi-Synthelabo and Roche Pharmaceuticals. Drug acquisition costs were obtained from the British National Formulary (Joint Formulary Committee, 2005). The costs of hospital attendances and follow-up tests were derived from the NHS Reference Costs (Department of Health, 2005). Pharmacy costs were obtained through personal communication (Michelle Rowe, The Christie Hospital NHS Trust, 2005). The costs of diagnostic tests and imaging were obtained from the literature (Mant and Primrose, 2004; Renehan *et al*, 2004).

The costs associated with relapse were taken from a recent health economic evaluation of irinotecan and oxaliplatin for the treatment of metastatic CRC undertaken on behalf of NICE (Hind *et al*, 2005). In line with NICE guidance at the time of the analysis, the model assumes that patients who relapse would be offered first-line 5-FU/LV, followed upon progression by second-line single-agent irinotecan (National Institute for Clinical Excellence, 2002). The impact of the expected costs and health outcomes resulting from the use of more recently recommended regimens of irinotecan, oxaliplatin and 5-FU/LV (based on our analysis of data from the GERCOR trial by Tournigand *et al*, 2004) for the treatment of metastatic CRC were explored within the sensitivity analysis. Further details of all cost parameters are available within the full study report (Pandor *et al*, 2005). All costs were valued at 2004 prices and discounted at a rate of 6%.

### Sensitivity analysis

A number of one-way sensitivity analyses were undertaken to explore the impact of alternative parametric assumptions on the central estimates of cost-effectiveness. These included alternative assumptions concerning discount rates for costs and health outcomes, alternative utility scores for patients who suffer a relapse and for those who remain disease-free, and assumptions concerning the impact of recent changes to guidance on the use of cytotoxic regimens for metastatic disease. Structural sensitivity analyses were also undertaken to explore the impact of alternative durations over which patients may relapse on cost-effectiveness estimates, as well as the impact of assuming a 5-year time horizon for the evaluation of costs and health outcomes.

Probabilistic sensitivity analysis was undertaken to generate information on the probability that each intervention produces the greatest level of net benefit (Claxton *et al*, 2005). Each model parameter was assigned a unique probability distribution based upon published estimates of uncertainty. This joint uncertainty was then propagated through the model using Monte Carlo sampling techniques to generate distributions of lifetime costs and health outcomes for patients receiving each treatment regimen. The results of the probabilistic sensitivity analysis are presented as marginal cost-effectiveness planes. Further details of the modelling methods and data sources used within this study are available from the full study report (Pandor *et al*, 2005).

## RESULTS

### Central estimates of cost-effectiveness

Table 2 presents the expected costs and health outcomes for each of the four interventions. Estimates of discounted LYGs are shown in parentheses.

Table 2 suggests that capecitabine is expected to result in cost-savings of approximately £3320 per patient in comparison with the Mayo Clinic 5-FU/LV regimen, while also providing an additional 0.98 QALYs over the 50-year model time horizon; in other words, capecitabine is expected to dominate 5-FU/LV. FOLFOX4 is estimated to produce an additional 1.32 QALYs at an additional cost of £3941 in comparison with the de Gramont 5-FU/LV regimen; the marginal cost-effectiveness of FOLFOX4 versus the de Gramont 5-FU/LV regimen is estimated to be £2970 per additional QALY gained.

Importantly, the MOSAIC trial used the de Gramont 5-FU/LV regimen as the control arm. While this regimen is not commonly used in the adjuvant treatment of colon cancer in the UK, direct evidence suggests that bolus and infusional 5-FU/LV are similar in terms of overall survival, albeit with fewer adverse events associated with the infusional regimens. If the Mayo Clinic regimen is assumed to have the same efficacy as the De Gramont regimen, the mean incremental cost-effectiveness of FOLFOX4 compared with the Mayo Clinic regimen would be around £10 000 per QALY gained. Similarly, such indirect comparisons suggest that FOLFOX4 *vs* capecitabine is expected to cost approximately £12 874 per additional QALY gained. However, as there are no RCTs, which have included either of these comparisons in the adjuvant treatment of stage III colon cancer, conclusions based upon such indirect comparisons should be approached tentatively.

### Uncertainty analysis

#### One-way and structural sensitivity analysis

Table 3 presents the results of the one-way and structural sensitivity analysis.

The sensitivity analysis suggests that the central estimates of cost-effectiveness are robust to changes in individual parameter values. The model suggests that capecitabine is consistently expected to dominate 5-FU/LV (Mayo Clinic regimen), and that the marginal cost-effectiveness of FOLFOX4 versus 5-FU/LV (de Gramont regimen) is no higher than approximately £17 000 per QALY gained, even when no further benefits are assumed to accrue beyond the duration of the MOSAIC trial.

#### Probabilistic sensitivity analysis

**Figure 2:**
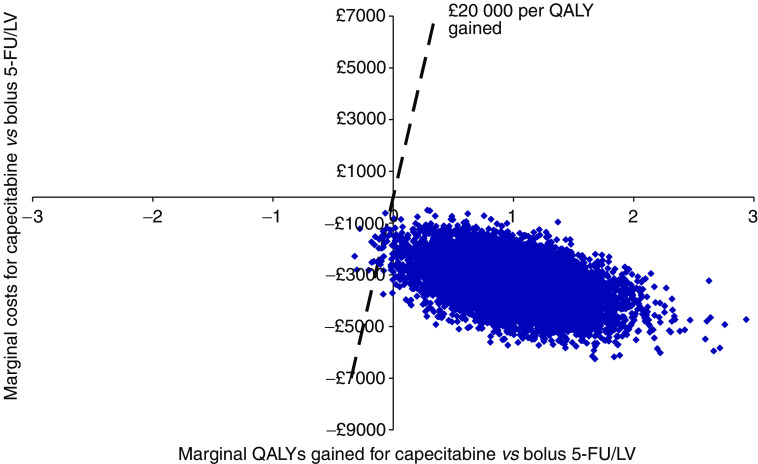
Cost-effectiveness plane for capecitabine *vs* 5-FU/LV (Mayo clinic regimen).

Figure 2 presents a cost-effectiveness plane showing the marginal costs and QALYs associated with capecitabine in comparison to the Mayo Clinic 5-FU/LV regimen.

Figure 2 suggests that capecitabine is consistently expected to result in cost-savings and provide additional health gains when compared to bolus 5-FU/LV. The uncertainty analysis suggests that the probability that capecitabine has a marginal cost-effectiveness that is better than £20 000 per QALY gained is estimated to be approximately 0.998 when compared to 5-FU/LV.

**Figure 3:**
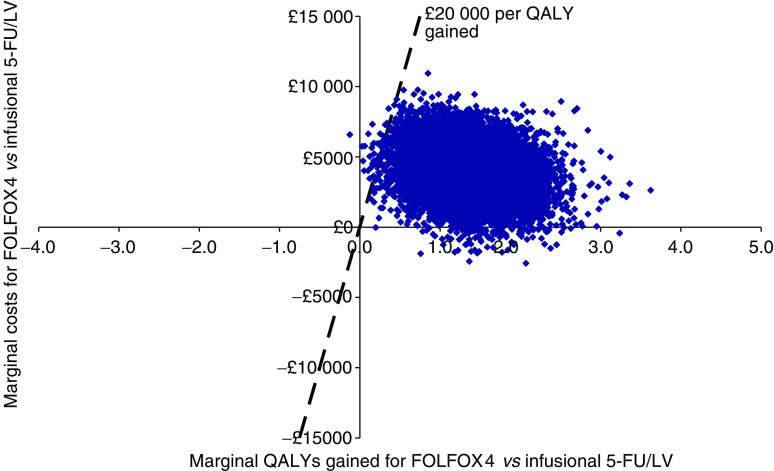
Cost-effectiveness plane for FOLFOX4 *vs* 5-FU/LV (de Gramont regimen).

Figure 3 presents a marginal cost-effectiveness plane describing the marginal costs and QALYs gained for FOLFOX4 *vs* the de Gramont 5-FU/LV regimen.

Figure 3 suggests that FOLFOX is expected to produce greater health gains than 5-FU/LV albeit at a greater cost. The probability that FOLFOX has a marginal cost-effectiveness that is better than £20 000 per QALY gained is estimated to be approximately 0.997 when compared to 5-FU/LV.

## DISCUSSION

The health economic analysis presented within this study suggests that both capecitabine and FOLFOX4 are expected produce health gains at a cost which is currently considered acceptable to the NHS in England and Wales (NICE, 2004b; NICE, 2006). The health economic model suggests that capecitabine is expected to be more effective and less expensive than 5-FU/LV, while the marginal cost-effectiveness of FOLFOX4 versus 5-FU/LV is estimated to be below £3000 per QALY gained. As with any health economic model, the data used to inform the analysis is subject to uncertainty and assumptions are required in the absence of empirical clinical evidence. However, the sensitivity analyses demonstrate that capecitabine is consistently expected to dominate the Mayo 5-FU/LV regimen, irrespective of assumptions concerning discount rates, utility scores as well as structural assumptions concerning the natural history of the disease (see Table 3). The marginal cost-effectiveness of FOLFOX4 *vs* 5-FU/LV also remains favourable when conservative parameter values are assumed.

Very few previous studies have attempted to estimate the cost-effectiveness of capecitabine and oxaliplatin plus 5-FU/LV in the adjuvant setting. Douillard *et al* (2004) conducted an economic analysis of capecitabine using data from the X-ACT study, and concluded that capecitabine would save an average of £1864 per patient compared with 5-FU/LV, and would offer a survival benefit of 8.7 additional quality-adjusted life-months (approximately 0.73 additional QALYs). This study used different assumptions concerning relapse-free and OS benefits beyond the duration of the trial. Koperna and Semmler (2003) reported a cost per LYG of €12 485 (approximately £8500) per LYG for oxaliplatin in combination with 5-FU/LV *vs* 5-FU/LV. However, this study used data from trials of oxaliplatin in patients with metastatic colorectal cancer as the basis for estimating survival outcomes for patients with stage III disease in the adjuvant setting. Aballea *et al* (2005) conducted an economic analysis of FOLFOX4 and 5-FU/LV using data from the MOSAIC trial, and reported a cost per LYG of US $27 300 (approximately £14 560). As this study has been published only in abstract form, it is difficult to pinpoint the key differences between our model and the economic analysis reported by Aballea *et al*. It is reasonable to suggest that these differences are a result of the omission of health-related quality of life information, the use of equivalent discount rates for both costs and health outcomes, and the adoption of a US Medicare perspective.

Importantly, there are limitations surrounding the use of evidence from the MOSAIC and X-ACT studies to inform policy decisions concerning clinically effective and cost-effective treatment options for patients with colon cancer in England and Wales. Notably, patients enrolled within these trials were comparatively younger than the typical CRC population treated on the NHS; patients in both studies had a mean age of around 60 years, while the median age of diagnosis of colon cancer is over 70 years (Cooper *et al*, 2005; Rowan *et al*, 2005; Welsh Cancer Intelligence and Surveillance Unit, 2005), hence the long-term survival benefits associated with each intervention may have been overestimated within the model. This bias may potentially result in optimistic marginal cost-effectiveness estimates for both FOLFOX4 and capecitabine. Evidence from a meta-analysis of trials in the adjuvant setting (Matasar *et al*, 2004), which conducted separate analyses for patients aged 70 years or under and those aged above 70 years, suggests that there is no significant difference in either OS or DFS at 8 years post-randomisation. However, the distribution of patient ages within each group was not reported, and it is unclear whether the survival curves presented in the Matasar *et al* (2004) study included all-cause mortality within the DFS curves. It is also noteworthy that the long term impact of oxaliplatin plus 5-FU/LV on neuropathy and its associated relationship with health-related quality of life is not currently known; given the absence of evidence, this economic analysis dues not explicitly address this issue.

The key assumption employed within the health economic model is that the short-term survival benefits of capecitabine and FOLFOX4 translate into long-term health gains; in other words, benefits accrued in the short-term are assumed to be sustained in the long-term. The absence of empirical long-term evidence makes this assumption difficult to validate, hence survival modelling methods were required to estimate long-term disease outcomes. The accuracy of these methods will only become clear once long-term survival data from the X-ACT and MOSAIC studies becomes available. A recent analysis of data from over 20 000 patients in 18 randomised trials of FU-based adjuvant therapy concluded that a difference in 3-year DFS is a strong predictor of 5-year OS benefit, which would broadly support the assumptions made in our model (Sargent *et al*, 2005). However, it is possible that the incremental benefit of oxaliplatin could be lessened if oxaliplatin pretreatment affects subsequent treatment choices and/or efficacy in relapsed patients. Similarly, long-term neurotoxicity following oxaliplatin therapy could impact upon a patient's health-related quality of life and profoundly affect QALY gains, but this can only be assessed using long-term follow-up data.

The implications of the X-ACT and MOSAIC data for clinical decision-making are complex. In particular, while each trial has demonstrated clinical and cost benefit for the intervention under assessment (the substitution of capecitabine in place of 5-FU/LV; addition of oxaliplatin) the trials do not clarify whether one or other of the two, or both, interventions should be adopted for maximum benefit. Evidence from further trials, including trials using oxaliplatin/capecitabine combinations may help to clarify these issues. Meanwhile, since oxaliplatin plus capecitabine is not currently licensed in the adjuvant setting, oncologists will need to evaluate the costs and benefits of the available treatment options for individual patients.

## Figures and Tables

**Table 1:** Chemotherapy regimens included in the health economic model

**Trial**	**Regimen**	**Cycle length (weeks)**	**Cycles per treatment course**	**Total protocol dose per cycle**
MOSAIC	Oxaliplatin+5-FU/LV	2	12	800 mg m^−2^ bolus 5-FU 1200 mg m^−2^ infusional 5-FU 400 mg m^−2^ leucovorin 85 mg/m^2^ oxaliplatin
	5-FU/LV (de Gramont regimen)	2	12	800 mg m^−2^ bolus 5-FU 1200 mg m^−2^ infusional 5-FU 400 mg m^−2^ leucovorin
X-ACT	Capecitabine	3	8	35 000 mg m^−2^ capecitabine
	5-FU/LV (Mayo Clinic regimen)	4	6	2125 mg m^−2^ bolus 5-FU 100 mg m^−2^ leucovorin

FU, fluorouracil; LV, leucovorin.

**Table 2:** Summary of cost-effectiveness results

**Adjuvant treatment option**	**Mean discounted QALYs (LYGs)**	**Mean discounted costs**
5-FU/LV (Mayo Clinic)	8.47 (9.87)	£13 239
Capecitabine	9.45 (10.88)	£9919
Difference	0.98 (1.02)	−£3320
Marginal cost per QALY gained (capecitabine versus Mayo Clinic regimen)	*Dominating*	
		
5-FU/LV (de Gramont)	9.39 (10.80)	£22 261
FOLFOX4	10.71 (12.15)	£26 202
Difference	1.33 (1.36)	£3940
Marginal cost per QALY gained (FOLFOX4 versus de Gramont regimen)	£2970	

FU=fluorouracil; LV=leucovorin; QALY=quality adjusted life year; LYG=life year gained.

**Table 3:** Sensitivity analysis results

**Parameter**	**Value in base**-**case analysis**	**Value in sensitivity analysis**	**Cost per QALY gained (capecitabine versus bolus 5-FU/LV)**	**Cost per QALY gained (FOLFOX4 versus infusional 5-FU/LV)**
Base case	—	—	*Dominating*	£2970
Discount rates for costs and health outcomes	6% for costs, 1.5% for health outcomes	3.5% for costs and health outcomes (NICE, 2004b)	*Dominating*	£3723
Discount rates for costs and health outcomes	6% for costs, 1.5% for health outcomes	Undiscounted	*Dominating*	£2364
Utility for patients with relapse	0.24 (Ness *et al*, 1999)	0.575 (Petrou and Campbell, 1997)	*Dominating*	£3069
Utility for patients with relapse	0.24 (Ness *et al*, 1999)	0.1 (Petrou and Campbell, 1997)	*Dominating*	£2930
Utility for patients without relapse	0.92 (Ramsey *et al*, 2000)	0.5 (assumption)	*Dominating*	£5584
Costs and outcomes for relapsers based on FOLFOX/FOLFIRI sequence	Cost=£9638 (Hind *et al*, 2005) QALYs=1.38 (Hind *et al*, 2005)	Cost=£21 742 (Hind *et al*, 2005) QALYs=2.28 (Hind *et al*, 2005)	*Dominating*	£1679
Costs and outcomes for relapsers based on FOLFIRI/FOLFOX sequence	Cost=£9638 (Hind *et al*, 2005) QALYs=1.38 (Hind *et al*, 2005)	Cost=£22 746 (Hind *et al*, 2005) QALYs=2.14 (Hind *et al*, 2005)	*Dominating*	£1565
Assumed duration over which relapses may occur^a^	5-years	10-years	*Dominating*	£1963
Time horizon^a^	50-years	Within-trial analysis (5-year horizon)	*Dominating*	£17 115

FU=fluorouracil; LV=leucovorin; QALY=quality adjusted life year.

a Structural sensitivity analysis.

**Box 1:** Key assumptions of the health economic model

• Survival following relapse is assumed to be independent of time of relapse and adjuvant treatment received (e.g. patients relapsing after FOLFOX4 are assumed to still have the same treatment options and expected survival as those relapsing after 5-FU/LV). While earlier relapse may indicate more aggressive disease and a poorer prognosis, without patient-level data this assumption is inevitable
• The survival of patients following relapse is assumed to be equivalent to survival of patients enrolled within the FOCUS trial
• All relapses are assumed to occur within five years following resection of the primary tumour. Clinical evidence from long-term follow-up of patients undergoing adjuvant chemotherapy supports this assumption (Moertel *et al*, 1995). The impact of this assumption on the central estimates of cost-effectiveness was tested within the sensitivity analysis
• Patients with subsequent metastatic disease are assumed to receive first-line 5-FU/LV followed upon progression by single-agent irinotecan
• In line with the administration schedule used in the MOSAIC trial (André *et al*, 2004), patients receiving 5-FU/LV via the de Gramont regimen are assumed to receive their treatment on an outpatient basis
